# Harnessing Nanopore Sequencing to Investigate the Epigenomic Landscape in Molar Incisor Hypomineralization—A Pilot Study

**DOI:** 10.3390/ijms26073401

**Published:** 2025-04-05

**Authors:** Silvia Salatino, Piotr Cuber, Wojciech Tynior, Carla Gustave, Dorota Hudy, Yuen-Ting Chan, Agnieszka Raczkowska-Siostrzonek, Raju Misra, Dagmara Aleksandrowicz, Dariusz Nałęcz, Joanna Katarzyna Strzelczyk

**Affiliations:** 1 Molecular Biology Laboratories, Science and Innovation Platforms, Natural History Museum, London SW7 5BD, UK; 2 Department of Medical and Molecular Biology, Faculty of Medical Sciences in Zabrze, Medical University of Silesia in Katowice, 41-808 Zabrze, Poland; 3 Department of Dental Surgery, Faculty of Medical Sciences in Zabrze, Medical University of Silesia in Katowice, 40-055 Katowice, Poland; 4 Public Health Microbiology, United Kingdom Health Security Agency, London E14 4PU, UK; 5 Department of Otolaryngology and Maxillofacial Surgery, St. Vincent De Paul Hospital, 81-348 Gdynia, Poland

**Keywords:** molar incisor hypomineralization, dental enamel, epigenomics, DNA methylation, nanopore sequencing, DNA transposable elements

## Abstract

Molar incisor hypomineralization (MIH) is a dental condition that affects the enamel of permanent molars and/or incisors, often leading to tooth decay. Although several etiological hypotheses have come forward, including prenatal medical problems and postnatal illness, the pathogenesis of MIH is yet unclear. Aimed at exploring the epigenomic landscape of this dental condition, we collected dental tissue from a MIH-affected child and an age-matched control patient and investigated their DNA methylation status through an in-depth analysis of nanopore long-read sequencing data. We identified 780,141 CpGs with significantly different methylation levels between the samples; intriguingly, the density of these dinucleotides was higher in the regions containing genes involved in dental morphogenesis and inflammatory processes leading to periodontitis. Further examination of 54 genes associated with MIH or hypomineralized second primary molar disorders revealed very distinct methylation of intragenic transposable elements (SINEs, LINEs, and LTRs), while functional profiling analysis of 571 differentially methylated regions genome-wide uncovered significant enrichment processes including ameloblasts differentiation and calcium ion binding, as well as SP1 and other zinc finger transcription factors. Taken together, our findings suggest that DNA methylation could play a role in the pathogenesis of MIH and represent a stepping stone towards a comprehensive understanding of this multifactorial disorder.

## 1. Introduction

Molar incisor hypomineralization (MIH) is a developmental defect affecting the enamel tissue of permanent teeth and has a pooled prevalence of 13.5% [1]. A systematic review by Pentapati et al. [2] found that some studies reported a higher prevalence of MIH among girls, while others found no significant difference between genders. Dental practitioners can diagnose MIH when at least one permanent first molar shows signs of the condition [3], although this disorder can also occur on incisors. The severity of the condition can vary significantly. Clinicians may observe a range of demarcated opacities in the affected teeth (including white, creamy, yellow, and brown shades), post-eruptive enamel breakdown, atypical restorations, and lack of MIH-affected teeth due to tooth extraction [4]. Early detection and prompt diagnosis of MIH are critical, as the condition can significantly affect children’s oral health by accelerating the development of caries in affected teeth. There is considerable interest in the etiology of MIH, which remains poorly understood due to the complex pathogenesis resulting from the interaction of multiple factors [5]. Five main groups of factors contributing to MIH development have been identified: environmental influences, perinatal conditions, past illnesses, administered medications, and genetic background [6]. Difficulties in determining a definitive etiology arise from methodological variability in studies, small sample sizes, and the interference between potential etiological factors. Garot et al. [5] conducted a systematic review with meta-analysis on MIH etiological factors, analyzing 45 studies worldwide. Factors were classified as prenatal, perinatal, or postnatal. Maternal illness was identified as a prenatal risk factor. Significant perinatal factors included birth hypoxia, cesarean delivery, and prematurity. Postnatally, MIH risk increased with antibiotic use and several systemic pathologies, including measles, urinary tract infection, otitis media, gastric disorders, bronchitis, kidney diseases, pneumonia, and asthma. Current knowledge indicates that genetic and/or epigenetic factors may also exert strong effects [7,8]. Special attention is given to genes involved in amelogenesis. These genes can be involved with the clinical manifestation of the disease and teeth colour and opacity. There are also studies concerning immune response genes, which play a vital role in protecting against oral pathogens and are therefore an active area of research in dental and medical genetics [9]. In our previous research, we performed a pioneering quantitative study of the global methylation level, using a 5-mC DNA ELISA kit, in samples of buccal mucosa epithelium taken from a cohort of MIH-affected children, and compared these to a control group of healthy individuals. Although there was no statistically significant difference between the two groups, our observations confirmed the association between epigenetic changes and various prenatal, perinatal, and postnatal factors [10]. In the current study, we focused more directly on dental tissue material and performed an in-depth, gene-level explorative analysis of the DNA methylation status from a MIH-affected child and a matched healthy control, using Oxford Nanopore sequencing technology.

## 2. Results

### 2.1. Genome-Wide Distribution of Methylation Events

**Figure 1:**
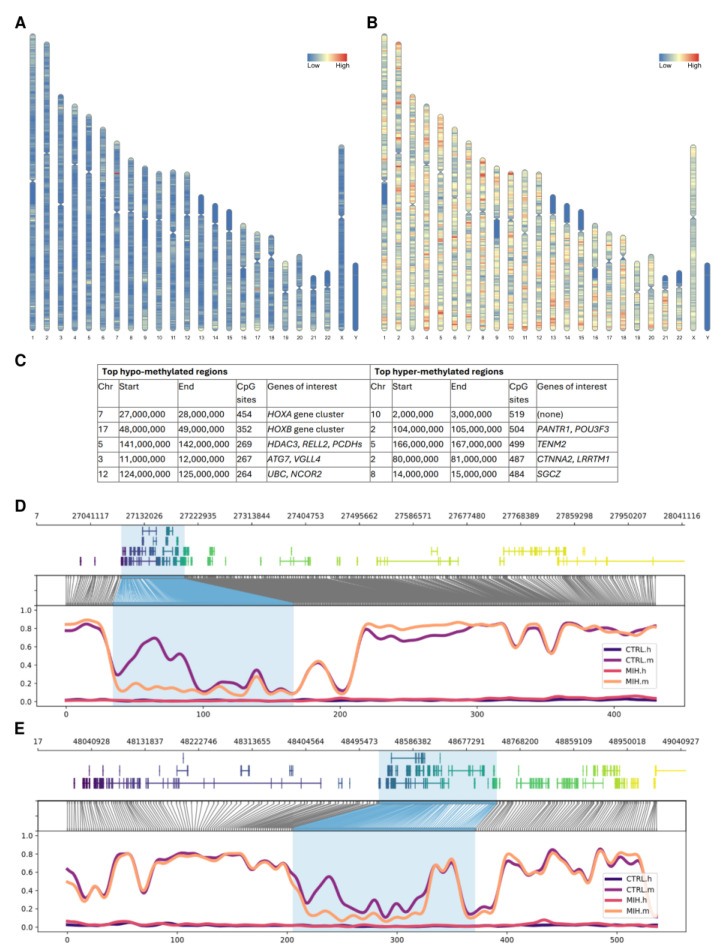
Chromosome ideograms showing the genomic distribution of significantly different hypo-methylated (**A**) and hyper-methylated (**B**) CpG sites identified in the MIH sample compared to the control sample. The top five 1 Mb windows hypo- and hyper-methylated in the MIH sample compared to the control sample (**C**). Methylation profile plots of the first (**D**) and second (**E**) hypo-methylated windows, showing (from top to bottom panel): genomic coordinates, gene models (coloured used the viridis palette for ease of reading), translation from genome to CpG-only coordinate space, and smoothed methylated fraction plot. The *HOXA* and *HOXB* gene clusters are highlighted in blue. The “_m” and “_h” suffix in sample names indicate the methyl-CpG (mCpG) and hydroxymethyl-CpG (hmCpG) signal, respectively.

Long-read nanopore sequencing of the control and MIH samples resulted in 19,469,702 reads (with mean read length 2369.1 bp, longest read length 1,452,954 bp, N50 read length 5981 bp, and median read quality 12.7 Q) and 13,534,170 reads (with mean read length 5660.4 bp, longest read length 1,313,331 bp, N50 read length 12,655 bp, and median read quality 12.9 Q), respectively. The global methylation levels were similar across the two samples (72% for control and 76% for MIH), and within the range of 70–80% CpG dinucleotides that have been observed to be stably methylated in mammalian cells [11]. Out of the ~28 million CpG sites present in the human genome, 780,141 had different methylation levels between the control and MIH samples that were statistically significant (*p* < 0.05). Of these, 150,458 CpG dinucleotides were hypo-methylated in the MIH-affected child compared to the healthy control, whereas 629,683 were hyper-methylated. As shown in Figure 1A,B, hypo- and hyper-methylated CpGs were distributed across all chromosomes, with a lower density around the centromere of chromosomes 1, 9, and 16, on the long arm of chromosome Y, as well as on the short arms of chromosomes 13, 14, 15, 21, and 22, in line with the known AT-rich regions present in those segments. After closer investigation of the five most hypo- and hyper-methylated regions, identified using 1 Mb windows sorted on the basis of their content of CpG sites, we found them to be hosting several dental- and inflammation-related genes (see Figure 1C and Section 3 for more details), including the entire *HOXA* and *HOXB* gene clusters (Figure 1D,E). Reducing the window size down to 1000 bp to verify that these gene-to-CpG associations were not spurious did not have a major effect on the results as the majority of the genes of interest listed in Figure 1C were still consistently ranking highly.

### 2.2. Differences in Methylation Signal of Promoters and Transposable Elements Within MIH-Associated Genes

Next, we investigated whether there was any significant difference in the methylation signal at the individual gene level. To minimize the chance of spurious associations with MIH due to confounding factors, we decided to use a curated set of 54 genes from the meta-analysis study of da Silva Figueira et al. [9] (which can be found in Supplementary Table S1) known to be associated with molar incisor hypomineralization or hypomineralized second primary molar disorders.

**Figure 2:**
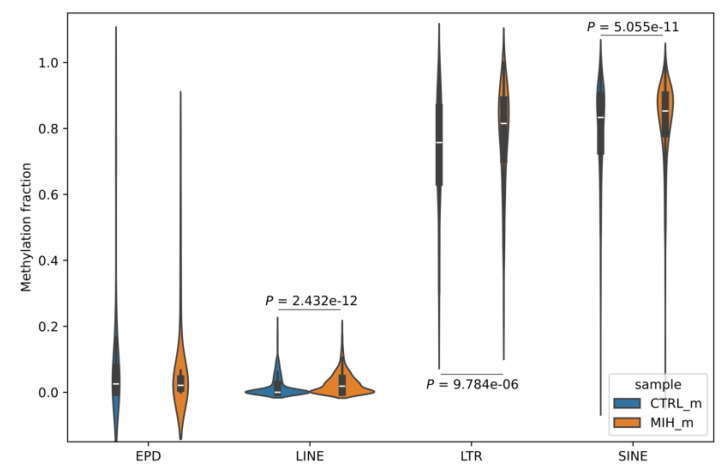
Violin plots representing the fraction of aggregated mCpG methylation signal in promoters (eukaryotic promoter database, EPD) and transposable elements (long interspersed nuclear elements, LINEs; long terminal repeats, LTRs; short interspersed nuclear elements, SINEs) overlapping the 54 genes of interest; P indicates the p-value, as calculated using the Wilcoxon signed-rank test.

**Figure 3:**
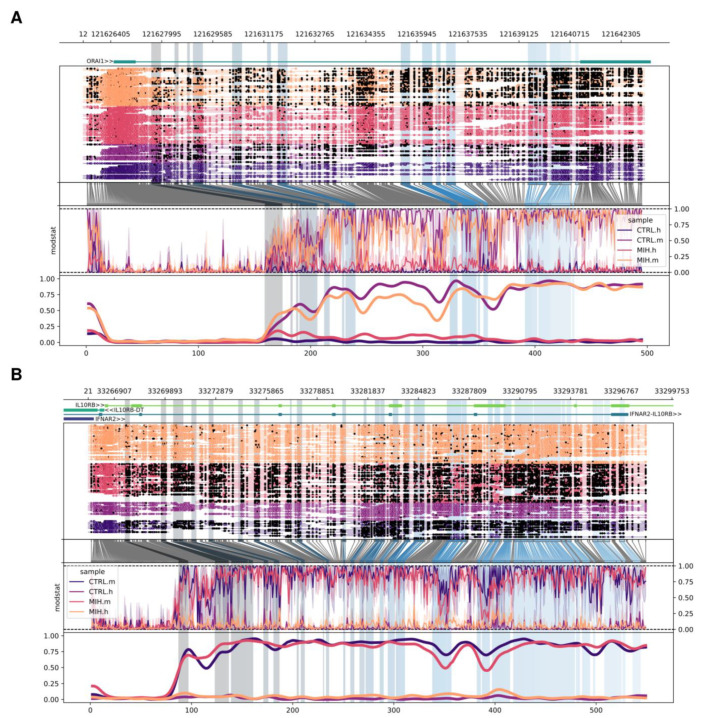
Methylation profiles of two genes ((**A**): *ORAI1*, (**B**): *IL10RB*) from the set of 54 genes associated with MIH, or hypomineralized second primary molar disorders. Tracks represent (from top to bottom panel): genomic coordinates, gene models (coloured used the viridis palette for ease of reading), read mappings with CpG bases as closed (modified) or open (unmodified) circles, translation from genome to CpG-only coordinate space, raw log-likelihood ratios, and smoothed methylated fraction plot. LINEs, LTRs, and SINEs are highlighted using a grey-to-blue colour palette.

To this purpose, we focussed on specific regions of interest that can affect gene regulation, including proximal promoters (downloaded from the EPDnew database and experimentally validated) and the three major families of transposable elements (TEs)—short interspersed nuclear elements (SINEs), long interspersed nuclear elements (LINEs), and long terminal repeats (LTRs). Although there was no significant difference in promoter regions, all intragenic transposable elements showed a highly significant difference between the methylation levels of the MIH and control sample (Figure 2). These results were validated using 10 different sets of 54 genes each, randomly sampled from all the human genes. Figure 3A,B show two examples of genes containing differentially methylated TEs and having different functions in the context of MIH; the calcium release-activated calcium modulator 1 (*ORAI1*) is a calcium protein channel that helps enamel-forming cells mineralize by regulating Ca^2+^ influx, and the interleukin 10 receptor subunit beta (*IL10RB*) is a cytokine involved in the inflammatory response of the tooth to caries [12].

### 2.3. Identification of Differentially Methylated Regions and Functional Profiling

**Figure 4:**
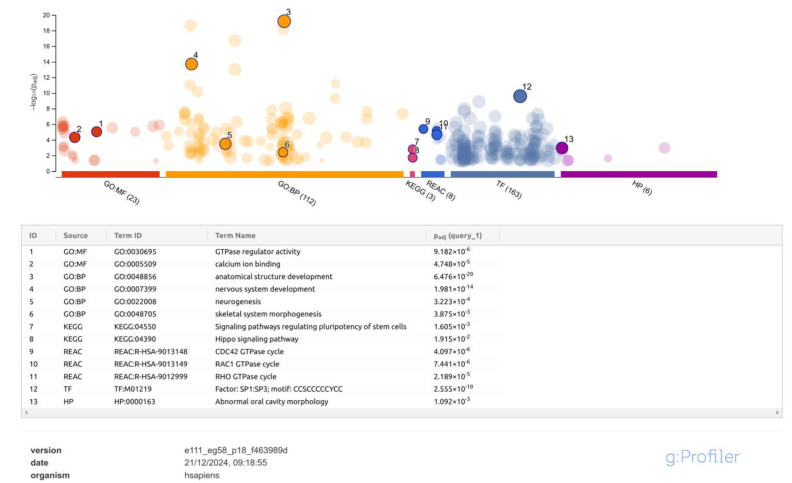
Significant ontology terms from functional profiling of the genes associated with the 571 DMRs (upper panel). Ontologies more closely related to MIH are circled, numbered, and listed in the lower panel, with term ID, term full name, and associated adjusted *p*-value. The full list of results can be found in Supplementary Table S3.

To investigate more systematically all the genes that might have been affected by methylation events, we first aggregated significant CpG sites into differentially methylated regions (DMRs). Then, we annotated the resulting 571 DMRs (Supplementary Table S2) to their closest genes within 100 bp and used this gene set as the input for functional profiling analysis. g:Profiler identified statistically significant enrichment for 23 terms from the molecular function sub-ontology in Gene Ontology (GO:MF), 109 terms from the biological process sub-ontology in Gene Ontology (GO:BP), 3 terms from the Kyoto Encyclopedia of Genes and Genomes (KEGGs) pathways, 8 terms from the reactome pathway database (REAC), 161 transcription factors from TRANSFAC (TF), and 8 terms from the human phenotype ontology (HP). A selection of the ontologies more closely related to MIH is shown in Figure 4 and commented on in Section 3, while the full list of results is provided in Supplementary Table S3.

## 3. Discussion

Epigenomics is a crucial yet understudied branch of biology. Recent work in this field demonstrated that epigenetic modifications, and DNA methylation changes in particular, are linked to and can lead to the development of a wide range of pathologies, including autoimmune disorders, cancers, and metabolic and neurological disorders [13]. With this project, we aimed to explore epigenomic changes in the context of the molar incisor hypomineralization (MIH) dental condition, through an in-depth, detailed examination of a case–control study.

Following the identification of 780,141 CpG dinucleotides with significantly different methylation levels between the control and MIH samples, we started to investigate the distribution of these methylation events along the genome and found that these tend to cluster in specific regions. The first two hypo-methylated regions, located on chr7:27,000,000–28,000,000 (p15.2–p15.1) and on chr17:48,000,000–49,000,000 (q21.32), host the so-called “*HOXA* gene cluster” and “*HOXB* gene cluster”, respectively, which are known to play an important role in development, including tooth morphogenesis [14]. The third window, located on chr5:141,000,000–142,000,000 (q31.3), contains the *HDAC3* gene, a key regulator of gingival fibroblast inflammatory responses in periodontitis [15] and *RELL2*, a homologue and co-regulator of the gene *RELT*, which causes hypomineralized amelogenesis imperfecta [16], and the protocadherins (*PCDHs*) gene cluster, a group of cell–cell adhesion molecules that play a role in odontogenesis [17] and are epigenetically regulated [18]. The fourth most hypo-methylated region, on chr3:11,000,000–12,000,000 (p25.3–p25.2) contains the *ATG7* gene, which is required for the secretion of iron from ameloblasts, the epithelial cells that produce enamel [19], and *VGLL4*, which has already been found to be differentially methylated in children with carious lesions by Silva et al. [20]. Finally, the fifth top window, located on chr12:124,000,000–125,000,000 (q24.31), contains the *UBC* gene, which is involved in a number of cellular processes that are relevant to tooth development and oral health, including dental pulp innervation [21], and the nuclear receptor co-repressor 2 (*NCOR2*) gene, a key mediator of transcriptional silencing which can reduce the expression of genes that cause inflammation [22]. This finding is particularly interesting in the context of molar incisor hypomineralization (MIH) as it can cause dental inflammation and pain.

Among the top hyper-methylated regions, although the one with the highest CpG site counts does not contain any tooth-related genes, the second one, on chr2:104,000,000–105,000,000 (q12.1), hosts the gene *PANTR1*, found to be involved in molar development in murine dental niche cells [23], and *POU3F3*, which is essential for the normal development of a part of the mammalian upper jaw and molars [24]. The third window, on chr5:166,000,000–167,000,000 (q34), contains the *TENM2* gene, which may play a part in the molecular etiology of periodontitis [25]. The fourth most hyper-methylated region in the MIH sample, located on chr2:80,000,000–81,000,000 (p12), hosts *CTNNA2*, a gene constitutively expressed in normal oral mucosa [26] which was found to be differentially expressed in murine ameloblasts [27], and *LRRTM1*, which was associated with dental caries in human adults [28]. Finally, the fifth window contains the *SGCZ* gene, which was found significantly up-regulated in the dental follicle tissue of healthy children [29].

Upon further investigation of specific genomic features—namely, proximal promoters and transposable elements (LINEs, LTRs, and SINEs, which contribute, respectively, 25%, 13%, and 8% of all CpG dinucleotides in the human genome [30])—we found a highly significant difference between the methylation levels of transposable elements (TEs) in the MIH and control samples. Although it is well known that TEs are able to influence gene expression through DNA methylation-mediated epigenetic control [31], to the best of our knowledge this has never been studied in the context of MIH or hypomineralized second primary molar disorders. In addition, our approach to use long-read sequencing data helped align these interspersed repeats—which typically range in length from 100 to 10,000 base pairs but can sometimes be far larger—with greater accuracy than the traditional short-read approach, thus providing more reliable results.

The results of our functional profiling analysis provided interesting insights about biological pathways related to MIH. Significant terms included processes known to be involved in the differentiation of ameloblasts and odontoblasts like the RhoA and Rac1 GTPase cycles [32], as well as calcium ion binding, which is crucial for proper enamel mineralization, and the Hippo pathway, which is essential for teeth development and morphogenesis [33] and also plays a critical role in endodontics, orthodontics, and periodontal diseases [34]. Intriguingly, significant ontology terms also included “nervous system development” and “signalling pathways regulating pluripotency of stem cells”, which suggest possible dysregulation of the dental pulp and nerve formation processes during childhood. This is further supported by the fact that the most enriched transcription factor was SP1, which has recently been shown to regulate the *KLF4* gene through DNA methylation during odontoblastic differentiation of human dental pulp cells [35]. In addition to SP1, Zhao et al. have recently revealed a novel protein network of transcription factors operating in the neural crest-derived dental mesenchyme [36]. This network regulating the complex odontogenesis process also includes factors encoded by the genes *MSX1*, *LHX6*, and *PAX9*, which we have shown to have a differentially methylated region nearby, thus suggesting that their dysregulation might contribute to the onset of MIH.

One of the major strengths of this work resides in its unique and innovative approach to adopt nanopore sequencing for investigating epigenomic changes in the context of MIH. Long-read technology allowed us to detect interspersed repeats of several kilobases in length and to align them to the reference genome with greater accuracy than traditional short-read approaches.

The main limitations of this study are the number of analyzed samples, namely one MIH-affected patient and a matching healthy child, and the age difference between them. Although we aimed to match the children as closely as possible to minimize the age discrepancy, stringent clinical criteria, such as the type of tooth (only first permanent molars) and its severe condition requiring extraction, restricted the available options only to a 9-year-old patient and to a 15-year-old control. Thus, we are aware that our results might have been affected by the bias likely introduced by the age gap between the individuals. Regarding the resolution of the data, although it was enough to enable us to explore the differences in the epigenomic landscape of the two individuals, a follow-up study on a larger cohort with better age-matched patients and with even higher sequencing depth can be envisioned to generalize our findings and draw more comprehensive conclusions.

## 4. Materials and Methods

### 4.1. Ethical Considerations

The Medical Ethics Committee of the Medical University of Silesia in Katowice, Poland, approved the study protocol (PCN/CBN/0022/KB1/108/IV/19/20/21/22, PCN/CBN/0052/KB1/145/21/22). After providing the parents or legal guardians with detailed information regarding the study’s objectives, written informed consent was obtained from them.

### 4.2. Clinical Examination

The criteria for identifying MIH were established by the European Academy of Pediatric Dentistry (EAPD). Dental professionals were familiarized with the EAPD guidelines and trained to minimize the risk of misdiagnosis. An interview and dental examination, including an evaluation of tooth morphology, were performed. A detailed examination of the children revealed no congenital defects, cranio-mandibular disorders, or genetic disorders affecting the mouth and teeth. There were no signs of common infectious diseases. They were not taking any medications, either permanently or temporarily. Two children who met the inclusion/exclusion criteria (listed in Table 1) were enrolled in the study. Detailed clinical characteristics of these children are presented in Table 2.

### 4.3. Sample Collection

**Figure 5:**
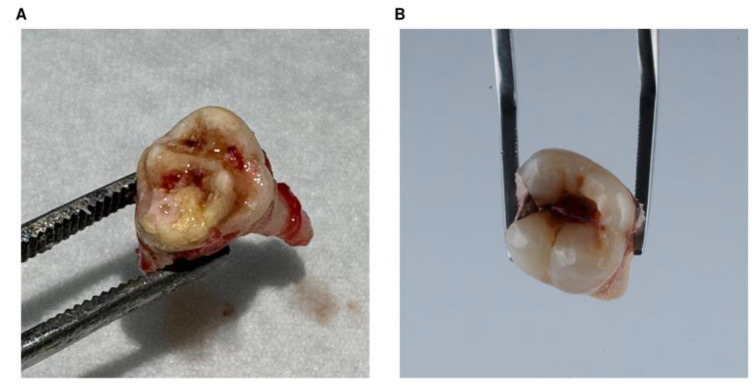
Photos of the MIH-affected tooth (**A**) and of the control tooth (**B**) from the matched healthy individual. Both teeth were first right permanent mandibular molars (FDI #46).

Dentists used a standard tooth extraction procedure with local anesthesia. The tissue sampling protocol required collecting tooth samples immediately after extraction (Figure 5) and preventing their degradation by storing them at sub-zero temperatures until further laboratory testing.

### 4.4. DNA Isolation from Teeth

Before DNA isolation, the teeth were pulverized with a mortar and pestle in liquid nitrogen. The isolation of DNA was performed with a GeneMATRIX bone DNA purification kit (Eurx, Gdańsk, Poland, #E3560-01) according to the manufacturer’s instructions. The concentration and purity of the DNA was determined using spectrophotometry in a NanoPhotometer Pearl UV/Vis Spectrophotometer (Implen, Munich, Germany). After isolation, it was stored at −20 °C. Transport for further analysis was conducted using dry ice.

### 4.5. ONT Sequencing

After transportation, the extracts were stored in −80 °C until further processing. In addition to spectrophotometric QC, the extracts were QC’d prior to sequencing via a Qubit fluorometer (ThermoFisher, Waltham, MA, USA) using a dsDNA HS kit (ThermoFisher) according to the attached protocol; DNA concentrations were 253 ng/µL for the control sample and 119 ng/µL for the MIH sample. Genomic tape and reagents (Agilent, Santa Clara, CA, USA) were used to measure the DNA fragment length and DIN values of the extracted DNA via the Tapestation 4200 instrument (Agilent) according to the attached protocol; the results were 7.1 for the control sample and 8.8 for the MIH sample. Libraries were prepared following the “Native barcoding genomic DNA” protocol, using a Ligation kit (SQK-LSK109) and a Native Barcoding Expansion kit 13–24 (EXP-NBD114) from Oxford Nanopore Technologies (Oxford, UK). Samples were sequenced in triplicates using PromethION R9 flow cells on a P2solo device using MinKNOW software version 24.02.16. (Oxford Nanopore Technologies). Each run/flow cell involved the control (healthy tissue) and the corresponding MIH sample, barcoded with separate barcodes (25 and 26 for teeth extracts). Each run lasted 72 h.

### 4.6. Bioinformatic Data Analysis

ONT reads from multiple sequencing runs were combined using Bash commands into two single samples, named “Tooth_CTRL” and “Tooth_MIH”, which then underwent an initial QC step to inspect read distribution and overall quality using NanoPlot [37], version 1.43.0, with options “--huge --no_static --N50”. Then, modBAM files generated by the software Dorado (Oxford Nanopore Technologies), version 7.2.13, within MinKNOW™ during ONT live basecalling were converted to FASTQ format using samtools [38], version 1.20, to preserve the “MM” and “ML” tags, and then passed as an input to minimap2 [39], version 2.28-r1209, with options “-ay --secondary=no -x map-ont”, to be mapped against the Ensembl Homo sapiens reference genome (assembly GRCh38). Next, the ONT software modkit (Oxford Nanopore Technologies), version 0.3.2, was used to first create summary counts of modified and unmodified bases in bedMethyl format, with options “pileup --cpg --combine-mods --combine-strands”, and later to perform differential methylation detection across the two samples at single-base genomic positions (that is, all the CpGs in the human genome), using options “dmr pair --base C --header”. Awk programming language, version 5.1.0, was used to select CpG sites with a minimum coverage of five reads in both samples and with a “map_pvalue” lower than 0.05. The R package RIdeogram [40], version 0.2.2, was used to draw SVG graphics to visualize and map genome-wide CpG data on chromosome ideograms. Methylation profiles for data visualization were generated using the “region” and “locus” commands in Methylartist [41], version 1.3.1. Promoter regions were downloaded from the Eukaryotic Promoter Database, EPDnew version 006 for hg38 (https://epd.expasy.org/epd/, last accessed 20 December 2024). Transposable elements were downloaded from the UCSC Table Browser, selecting the track dataset RepeatMasker for assembly GRCh38/hg38 (https://genome.ucsc.edu/cgi-bin/hgTables, last accessed 20 December 2024). The Wilcoxon signed-rank test was calculated for paired values of EPD, LINE, LTR, and SINE regions using the R base package (R Core Team. 2021. R: The R Project for Statistical Computing, https://www.r-project.org/), version 4.3.1. Significant CpG sites were aggregated into DMRs using Metilene [42], version 0.2-8, with default options except “--minMethDiff 0.2” (doubled, to identify regions with a substantial change in methylation levels), using the following criteria: (i) sequencing depth of each CpG site ≥ 5×; (ii) differential methylation of CpG sites between samples ≥ 0.2; (iii) number of differentially methylated CpG sites in the region ≥ 10; (iv) distance between adjacent differentially methylated CpG sites ≤ 300 bp; (v) Mann–Whitney U test with Bonferroni-adjusted *p*-value < 0.05. Gene annotations were added to the DMRs using bedtools [43] closest, version 2.30.0, with default options. Functional profiling was performed with g:Profiler [44], version e111_eg58_p18_f463989d, and using the following databases: hsapiens Ensembl genes version GRCh38.p14, GO release 2024-01-17, KEGG release 2024-01-22, Reactome release 2024-1-25, TRANSFAC release 2023.2, Human Phenotype Ontology release 01.2024; only ontology terms having an FDR-adjusted *p*-value of ≤0.05 were considered significant.

## 5. Conclusions

To the best of our knowledge, this is the first study to use Oxford Nanopore long-read sequencing to investigate DNA methylation changes in the context of MIH. We identified 780,141 CpG dinucleotides with different methylation levels between the control and MIH samples that were statistically significant. The genomic regions with the highest density of such CpGs contained a number of genes involved in dental morphogenesis and inflammatory processes leading to periodontitis. Further examination of a set of 54 genes associated with molar incisor hypomineralisation or hypomineralized second primary molar disorders revealed a highly significant difference between the methylation levels of intragenic transposable elements (LINE, LTR, and SINE), which are known to influence gene expression through DNA methylation-mediated epigenetic control, although there were no significant differences in the promoters of those genes. Finally, after aggregating differential CpG sites into 571 DMRs, a systematic functional profiling analysis of all the genes located close to those regions uncovered significant enrichment of terms related to ameloblast differentiation processes (like the RhoA and Rac1 GTPase cycles and the Hippo pathway) and calcium ion binding, as well as transcription factors like SP1, which mediates odontoblastic differentiation of human dental pulp cells. Overall, our findings indicate that DNA methylation might be involved in the pathogenesis of MIH and represent a step forward towards a thorough understanding of this complex disorder.

## Figures and Tables

**Table 1:** Inclusion and exclusion criteria used to select the MIH-affected patient and the control individual.

Inclusion Criteria	Exclusion Criteria
Age: 6–16	Birth defects
The condition of the first permanent molar did not allow for conservative and endodontic treatment	Genetic disorders
Parent or legal guardian agreement	Cranio-mandibular disorders and developmental defects of tooth (apart from MIH)

**Table 2:** Detailed clinical profiles of the two patients selected for the study.

		MIH	Control
Gender		female	female
Age		9	15
MIH TNI Index		4c	0
EAPD Criteria	Demarcated opacities	Present	No
	Post-eruptive enamel breakdown	Present	No
	Atypical restorations	Yes	No
	Extractions of molars due to MIH	No	No
	Failure of eruption of molar or an incisor	No	No

## Data Availability

Data available on request due to privacy and ethical restrictions.

## Supplementary Materials

The following supporting information can be downloaded at: https://www.mdpi.com/article/10.3390/ijms26073401/s1.

## References

[1] Lopes L.B., Machado V., Mascarenhas P., Mendes J.J., Botelho J. (2021). The Prevalence of Molar-Incisor Hypomineralization: A Systematic Review and Meta-Analysis. Sci. Rep..

[2] Pentapati K.C., Yeturu S.K., Siddiq H. (2017). Systematic Review and Meta-analysis of the Prevalence of Molar-incisor Hypomineralization. J. Int. Oral Health.

[3] Weerheijm K.L., Duggal M., Mejàre I., Papagiannoulis L., Koch G., Martens L.C., Hallonsten A.L. (2023). Judgement Criteria for Molar Incisor Hypomineralisation (MIH) in Epidemiologic Studies: A Summary of the European Meeting on MIH Held in Athens, 2003. Eur. J. Paediatr. Dent..

[4] Lygidakis N.A., Garot E., Somani C., Taylor G.D., Rouas P., Wong F.S.L. (2022). Best Clinical Practice Guidance for Clinicians Dealing with Children Presenting with Molar-Incisor-Hypomineralisation (MIH): An Updated European Academy of Paediatric Dentistry Policy Document. Eur. Arch. Paediatr. Dent..

[5] Garot E., Rouas P., Somani C., Taylor G.D., Wong F., Lygidakis N.A. (2022). An Update of the Aetiological Factors Involved in Molar Incisor Hypomineralisation (MIH): A Systematic Review and Meta-Analysis. Eur. Arch. Paediatr. Dent..

[6] Bekes K. (2022). Molar Incisor Hypomineralization.

[7] Bezamat M., Souza J.F., Silva F.M.F., Corrêa E.G., Fatturi A.L., Brancher J.A., Carvalho F.M., Cavallari T., Bertolazo L., Machado-Souza C. (2021). Gene-Environment Interaction in Molar-Incisor Hypomineralization. PLoS ONE.

[8] Silva M.J., Scurrah K.J., Craig J.M., Manton D.J., Kilpatrick N. (2016). Etiology of Molar Incisor Hypomineralization—A Systematic Review. Community Dent. Oral Epidemiol..

[9] da Silva Figueira R., Muniz F.W.M.G., Costa L.C., de Moura M.S., de Deys Moura L.D.F.A., de Oliveira B.M., Lima C.C.B., Rösing C.K., de Lima M.D.D.M. (2023). Association between Genetic Factors and Molar-Incisor Hypomineralisation or Hypomineralised Second. Primary Molar: A Systematic Review. Arch. Oral Biol..

[10] Tynior W., Ilczuk-Rypuła D., Hudy D., Strzelczyk J.K. (2022). Is Aberrant DNA Methylation a Key Factor in Molar Incisor Hypomineralization?. Curr. Issues Mol. Biol..

[11] Bird A., Taggart M., Frommer M., Miller O.J., Macleod D. (1985). A Fraction of the Mouse Genome That Is Derived from Islands of Nonmethylated, CpG-Rich DNA. Cell.

[12] Horst O.V., Horst J.A., Samudrala R., Dale B.A. (2011). Caries Induced Cytokine Network in the Odontoblast Layer of Human Teeth. BMC Immunol..

[13] Jin Z., Liu Y. (2018). DNA Methylation in Human Diseases. Genes Dis..

[14] Suryadeva S., Begum M. (2015). Role of Homeobox Genes in Tooth Morphogenesis: A Review. J. Clin. Diagn. Res..

[15] Lagosz K.B., Bysiek A., Macina J.M., Bereta G.P., Kantorowicz M., Lipska W., Sochalska M., Gawron K., Kaczmarzyk T., Chomyszyn-Gajewska M. (2020). HDAC3 Regulates Gingival Fibroblast Inflammatory Responses in Periodontitis. J. Dent. Res..

[16] Nikolopoulos G., Smith C.E.L., Brookes S.J., El-Asrag M.E., Brown C.J., Patel A., Murillo G., O’Connell M.J., Inglehearn C.F., Mighell A.J. (2020). New Missense Variants in RELT Causing Hypomineralised Amelogenesis Imperfecta. Clin. Genet..

[17] Heymann R., Kallenbach S., Alonso S., Carroll P., Mitsiadis T.A. (2001). Dynamic Expression Patterns of the New Protocadherin Families CNRs and Pcdh-γ during Mouse Odontogenesis: Comparison with Reelin Expression. Mech. Dev..

[18] El Hajj N., Dittrich M., Haaf T. (2017). Epigenetic Dysregulation of Protocadherins in Human Disease. Semin. Cell Dev. Biol..

[19] Sukseree S., Schwarze U.Y., Gruber R., Gruber F., Quiles Del Rey M., Mancias J.D., Bartlett J.D., Tschachler E., Eckhart L. (2020). ATG7 Is Essential for Secretion of Iron from Ameloblasts and Normal Growth of Murine Incisors during Aging. Autophagy.

[20] Silva M., Mohandas N., Craig J., Manton D., Saffery R., Southey M., Burgner D., Lucas J., Kilpatrick N., Hopper J. (2022). DNA Methylation in Childhood Dental Caries and Hypomineralization. J. Dent..

[21] Donnelly C.R., Shah A.A., Suh E.B., Pierchala B.A. (2019). Ret Signaling Is Required for Tooth Pulp Innervation during Organogenesis. J. Dent. Res..

[22] Paluvai H., Shanmukha K.D., Tyedmers J., Backs J. (2023). Insights into the Function of HDAC3 and NCoR1/NCoR2 Co-Repressor Complex in Metabolic Diseases. Front. Mol. Biosci..

[23] Hu H., Duan Y., Wang K., Fu H., Liao Y., Wang T., Zhang Z., Kang F., Zhang B., Zhang H. (2022). Dental Niche Cells Directly Contribute to Tooth Reconstitution and Morphogenesis. Cell Rep..

[24] Jeong J., Li X., McEvilly R.J., Rosenfeld M.G., Lufkin T., Rubenstein J.L.R. (2008). Dlx Genes Pattern Mammalian Jaw Primordium by Regulating Both Lower Jaw-Specific and Upper Jaw-Specific Genetic Programs. Development.

[25] Hong K.W., Shin M.S., Ahn Y.B., Lee H.J., Kim H.D. (2015). Genomewide Association Study on Chronic Periodontitis in Korean Population: Results from the Yangpyeong Health Cohort. J. Clin. Periodontol..

[26] Marconcini S., Denaro M., Cosola S., Gabriele M., Toti P., Mijiritsky E., Proietti A., Basolo F., Giammarinaro E., Covani U. (2019). Myofibroblast Gene Expression Profile after Tooth Extraction in the Rabbit. Materials.

[27] Miyata K., Chiba Y., Marchelina T., Inada S., Oka S., Saito K., Yamada A., Fukumoto S. (2023). Single-Cell RNA-Sequence of Dental Epithelium Reveals Responsible Genes of Dental Anomalies in Human. Pediatr. Dent. J..

[28] Laajala A., Pesonen P., Alaraudanjoki V., Anttonen V., Laitala M.L. (2023). Genome-Wide Association Study Identifies Novel Caries-Associated Loci Showing Sex-Specificity-A Study on the Northern Finland Birth Cohort 1966. Eur. J. Oral Sci..

[29] Lee H.S., Lee J., Kim S.O., Song J.S., Lee J.H., Lee S.I., Jung H.S., Choi B.J. (2013). Comparative Gene-Expression Analysis of the Dental Follicle and Periodontal Ligament in Humans. PLoS ONE.

[30] Luo Y., Lu X., Xie H. (2014). Dynamic Alu Methylation during Normal Development, Aging, and Tumorigenesis. Biomed. Res. Int..

[31] Gebrie A. (2023). Transposable Elements as Essential Elements in the Control of Gene Expression. Mob. DNA.

[32] Biz M.T., Marques M.R., Crema V.O., Moriscot A.S., Dos Santos M.F. (2010). GTPases RhoA and Rac1 Are Important for Amelogenin and DSPP Expression during Differentiation of Ameloblasts and Odontoblasts. Cell Tissue Res..

[33] Xiang L., Yu H., Zhang X., Wang B., Yuan Y., Zhang Q., Ye R., Gong P., Wu Y. (2018). The Versatile Hippo Pathway in Oral-Maxillofacial Development and Bone Remodeling. Dev. Biol..

[34] Ni D. (2024). The Hippo Pathway in Oral Diseases and Treatments: A Review. Medicine.

[35] Sun Z., Yu S., Chen S., Liu H., Chen Z. (2019). SP1 Regulates KLF4 via SP1 Binding Motif Governed by DNA Methylation during Odontoblastic Differentiation of Human Dental Pulp Cells. J. Cell Biochem..

[36] Zhao M., Gupta V., Raj L., Roussel M., Bei M. (2013). A Network of Transcription Factors Operates during Early Tooth Morphogenesis. Mol. Cell Biol..

[37] De Coster W., Rademakers R. (2023). NanoPack2: Population-Scale Evaluation of Long-Read Sequencing Data. Bioinformatics.

[38] Danecek P., Bonfield J.K., Liddle J., Marshall J., Ohan V., Pollard M.O., Whitwham A., Keane T., McCarthy S.A., Davies R.M. (2021). Twelve Years of SAMtools and BCFtools. Gigascience.

[39] Li H. (2018). Minimap2: Pairwise Alignment for Nucleotide Sequences. Bioinformatics.

[40] Hao Z., Lv D., Ge Y., Shi J., Weijers D., Yu G., Chen J. (2020). RIdeogram: Drawing SVG Graphics to Visualize and Map Genome-Wide Data on the Idiograms. PeerJ Comput. Sci..

[41] Cheetham S.W., Kindlova M., Ewing A.D. (2022). Methylartist: Tools for Visualizing Modified Bases from Nanopore Sequence Data. Bioinformatics.

[42] Jühling F., Kretzmer H., Bernhart S.H., Otto C., Stadler P.F., Hoffmann S. (2016). Metilene: Fast and Sensitive Calling of Differentially Methylated Regions from Bisulfite Sequencing Data. Genome Res..

[43] Quinlan A.R., Hall I.M. (2010). BEDTools: A Flexible Suite of Utilities for Comparing Genomic Features. Bioinformatics.

[44] Kolberg L., Raudvere U., Kuzmin I., Adler P., Vilo J., Peterson H. (2023). G:Profiler-Interoperable Web Service for Functional Enrichment Analysis and Gene Identifier Mapping (2023 Update). Nucleic Acids Res..

